# Valorization of Date Palm (*Phoenix dactylifera* L.) Fruits and By-Products as High-Value Sustainable Products: A Comprehensive Review on Bioactive Composition, Health Benefits, and Industrial Applications

**DOI:** 10.3390/molecules31071194

**Published:** 2026-04-03

**Authors:** Ouarda Djaoudene, Raquel Rodríguez-Solana, Anabela Romano

**Affiliations:** 1 Centre de Recherche en Technologies Agro-Alimentaires, Route de Targa Ouzemmour, Campus Universitaire, Bejaia 06000, Algeria; 2 Department of Agroindustry and Food Quality, Andalusian Institute of Agricultural and Fisheries Research and Training (IFAPA), Rancho de la Merced Center, Carretera Cañada de la Loba (CA-3102) Km 3.1., SN, Jerez de la Frontera, 11471 Cádiz, Spain; raquel.rodriguez.solana@juntadeandalucia.es; 3 MED—Mediterranean Institute for Agriculture, Environment and Development, CHANGE—Global Change and Sustainability Institute, Faculdade de Ciências e Tecnologia, Universidade do Algarve, Campus de Gambelas, 8005-139 Faro, Portugal

**Keywords:** date palm, date fruit by-products, nutritional compositions, functional ingredients, sustainable food systems, high-value products

## Abstract

Health-promoting foods are attracting growing interest as complements to pharmacological interventions, particularly when incorporated into bioactive-enriched functional foods. The date palm (*Phoenix dactylifera* L.) plays a key socio-economic role in arid and semi-arid regions, and is widely recognized for its high nutritional value, functional attributes, and therapeutic potential. Date fruits and their processing by-products, particularly the seeds, are a rich source of essential nutrients, dietary fiber, and diverse phytochemicals with documented antioxidant, anti-inflammatory, antidiabetic, and antimicrobial properties. This narrative review summarizes the latest evidence from experimental, preclinical, and emerging clinical studies on the nutritional composition, phytochemical profile, and biofunctional properties of dates and their derivatives, with particular emphasis on seeds as a significant processing by-product. Recent advances in their valorization for food applications, including bakery products, dairy products, beverages, meat products, confectionery, and active packaging, are critically discussed, as are their emerging uses in the pharmaceutical and related industries. Particular attention is given to their potential to improve the nutritional quality, functional performance, sensory attributes, and shelf life of food products. Overall, date fruits and their by-products are cost-effective, natural, and sustainable ingredients for developing value-added functional foods. Their efficient valorization offers promising strategies for reducing waste, implementing circular economy principles, and meeting the increasing consumer demand for healthier products. This review highlights the need for multidisciplinary research and innovation to advance sustainable by-product utilization, improve agro-industrial waste management, and expand the range of high-value applications for date fruits and seeds, thereby contributing to global food security, economic development, and improved public health.

## 1. Introduction

Plant-derived bioactive compounds are receiving increasing attention due to their potential applications in the food, cosmetic and pharmaceutical sectors. In this context, plant resources traditionally cultivated in arid and semi-arid regions are attracting renewed scientific interest. Among them, the date palm (*Phoenix dactylifera* L.) is a notable species with important nutritional, medicinal and industrial potential [1]. The preservation and sustainable exploitation of such plant resources can be achieved through the protection of genetic heritage and their economic valorization. These actions constitute key pillars of regional development strategies in arid and semi-arid zones [2,3,4].

**Figure 1:**
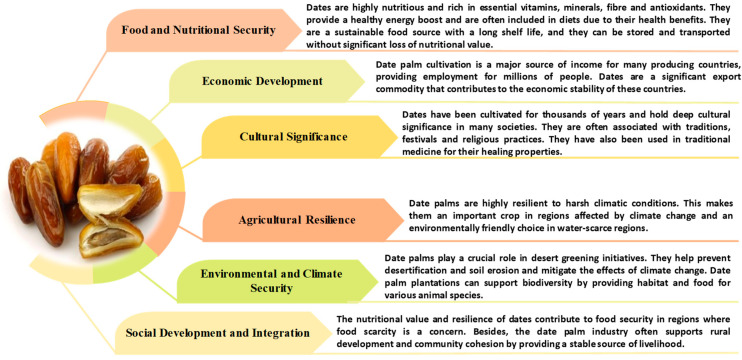
The global importance of date palm (*Phoenix dactylifera* L.) cultivation [8,33].

The date palm belongs to the *Arecaceae* family, which includes more than 200 genera and approximately 2500 species. More than 3000 cultivars of *P. dactylifera* have been identified worldwide and are commonly classified according to their geographical origin and fruit characteristics [5,6]. Date palm plays an important role in arid ecosystems by supporting food security and socio-economic development in many desert regions. Often referred to as the ‘tree of life’, the date palm provides edible fruit, animal feed, construction materials and a variety of traditional remedies [7,8]. As illustrated in Figure 1, this cultivation holds considerable global importance. 

**Figure 2:**
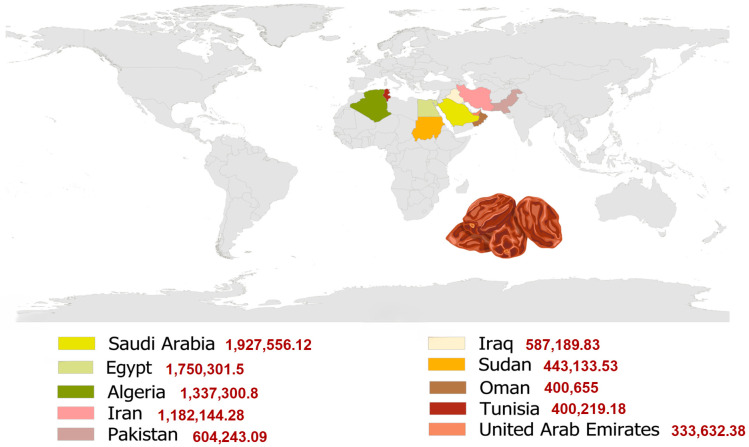
Top ten worldwide date-producing countries and their production (Tons) in 2024 [10].

Date palms are predominantly cultivated in arid and semi-arid climates, particularly in the Middle East and North Africa (MENA) region [9]. Global date production reached approximately 9.7 million tons in 2024, with major producers including Egypt, Saudi Arabia, Algeria, Iran, Pakistan, Iraq, Sudan, Oman, Tunisia and the United Arab Emirates (Figure 2) [10]. Reflecting this importance, the global dates market was valued at US$31.03 billion in 2024 and is projected to grow at a compound annual growth rate (CAGR) of 5.99% between 2025 and 2032, reaching approximately US$49.14 billion, with the Middle East and Africa accounting for more than 85% of global production [7,11,12,13].

Nutritionally, date fruits are characterized by high contents of dietary fiber, carbohydrates, essential minerals, vitamins, and unsaturated fatty acids, as well as a wide range of phytochemicals [14,15,16]. Increasing scientific evidence indicates that date fruits and their by-products, particularly date seeds, contain bioactive compounds such as phenolic acids, flavonoids and carotenoids. These compounds exhibit antioxidant, anti-inflammatory and antimicrobial properties. This suggests that they could play a role in preventing and managing chronic and infectious diseases [5,17,18,19,20,21]. Recent research has also highlighted the growing evidence supporting the therapeutic potential of *Phoenix dactylifera* L., which contains a wide range of bioactive constituents associated with beneficial effects [22,23,24]. However, although promising results have been reported, robust clinical evidence remains limited and further research is required to clarify the mechanisms of action and potential health benefits.

Currently, more than 5000 varieties of date palm are cultivated worldwide and are generally classified according to fruit texture, ripening time and commercial value [7,25]. Dates are consumed fresh or dried and are widely processed into value-added products such as syrups, pastes, sugars, jams, jellies, juices, vinegar and fermented products [8,14,26]. These processing activities generate substantial amounts of by-products, mainly seeds and pomace, which are often underutilized despite their high content of bioactive compounds and functional constituents.

In recent years, there has been an increasing focus on the valorization of these by-products through sustainable and green extraction technologies [27,28,29]. Several green extraction methods have been proposed as environmentally friendly alternatives for the efficient recovery of phenolic compounds and other bioactive molecules [30,31]. Furthermore, the valorization of date processing by-products is increasingly in line with the strategies of the circular bioeconomy, which aim to transform agricultural residues into high-value ingredients for use in the food, nutraceutical, cosmetic and pharmaceutical industries [8,26]. These approaches help to reduce agro-industrial waste, improve resource efficiency and promote more sustainable food production systems [32]. However, despite dates being widely consumed, the full nutritional, functional and medicinal potential of date fruits and their by-products remain largely unexplored.

Against this backdrop, this review aims to provide an updated, comprehensive and critical synthesis of current knowledge on date fruits and their processing by-products, with particular emphasis on date seeds as an underutilized resource. This narrative review integrates findings from experimental, preclinical, technological, and emerging clinical studies. It examines the nutritional composition, phytochemical profile, biofunctional properties, and industrial applications of date-derived materials. This work focuses on the valorization of both date fruits and seeds across multiple sectors. These include food, nutraceutical, cosmetic, and pharmaceutical applications within sustainability and circular bioeconomy frameworks. The analysis emphasizes recent advances in functional food development, and value-added product innovation, especially within the food sector. By emphasizing these applications, this review seeks to advance the sustainable exploitation of date palm resources within the broader context of global health, food security, and environmental challenges, while consolidating current knowledge and outlining strategic directions and priorities for future research.

## 2. Methodological Approach and Literature Search Strategy

This integrative narrative review was developed through a comprehensive qualitative synthesis of current scientific evidence on date fruits and their processing by-products, with particular emphasis on date seeds. A structured search strategy and transparent selection criteria were used to improve reproducibility and reduce selection bias. 

The literature searches were conducted in major scientific databases, including Scopus, Web of Science Core Collection, PubMed and Google Scholar, to ensure broad coverage of the available literature. The search focused on studies published between 2013 and 2026, with greater emphasis on studies published from 2020 onwards to reflect recent advances. Search terms were combined using Boolean operators and included: “date palm”, “*Phoenix dactylifera*”, “date fruit”, “date seeds” (or “date pits/kernels”), “by-products”, “bioactive compounds”, “phenolics”, “antioxidant”, “anti-inflammatory”, “antidiabetic”, “antimicrobial”, “valorization”, “functional foods”, “food applications”, “active packaging”, and “circular bioeconomy”. Eligible records were peer-reviewed articles directly addressing (i) nutritional composition and phytochemical profiling; (ii) biofunctional properties based on in vitro, in vivo, and/or human evidence; and/or (iii) technological/industrial applications in foods, packaging, or related sectors. Titles and abstracts were screened for relevance, followed by full-text assessment when necessary. Additional references were identified through citation tracking of key papers. Evidence was synthesized qualitatively and interpreted critically rather than quantitatively pooled. This approach was adopted due to the heterogeneity across cultivars, processing conditions, extraction methods, and study designs, as well as the limited availability of robust clinical trials.

## 3. Health Benefits Components of Date Palm

### 3.1. Nutritional Components

**Figure 3:**
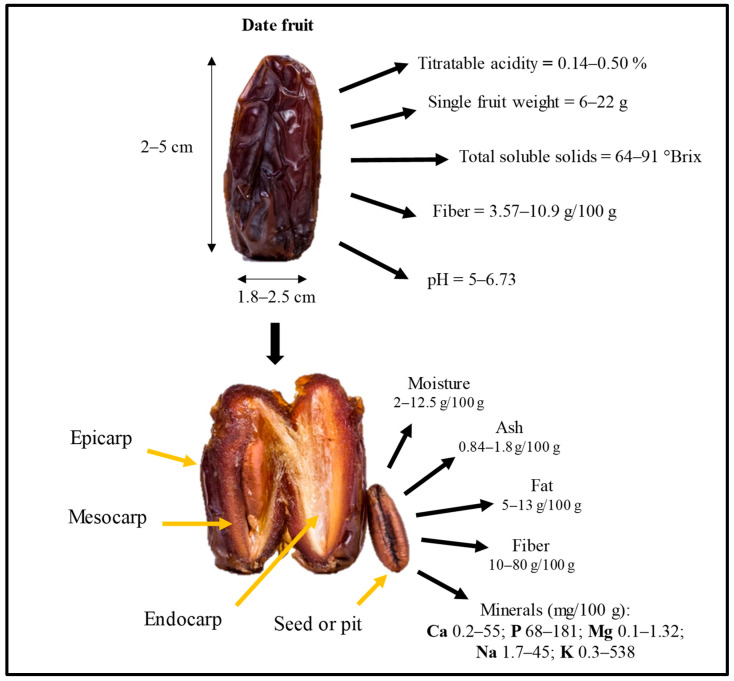
Morphology and chemical composition of date fruit and its seed [5,38,40,43].

The date fruit is a fleshy berry that can be either elongated or rounded in shape. It contains a single hard seed (see Figure 3). Its color can range from golden yellow to dark red or almost black, depending on the cultivar and stage of maturity. These morphological variations, frequently reported across studies, may influence compositional and functional attributes.

Dates are nutrient-rich fruits, composed mainly of edible pulp (85–90%) and a seed (10–15%). Their chemical composition varies depending on cultivar, geographical origin, harvest time and pre- and postharvest practices [5,12,14,26,34]. However, reported compositional ranges across studies are often difficult to compare because the data are expressed on different bases (fresh vs. dry weight), the fruit is at different stages of maturity, and the analytical protocols are diverse. Furthermore, cultivar/terroir effects are not consistently controlled. Consequently, broad ranges should be interpreted as indicative rather than being directly transferred to nutritional labeling or formulation targets. 

Dates are a rich source of essential macronutrients and micronutrients, with carbohydrates making up over 80% of their dry matter. These include monosaccharides and disaccharides such as glucose, fructose, mannose, galactose, maltose and sucrose, as well as minor amounts of polysaccharides such as cellulose, starch and β-glucans [6,35]. At full maturity, glucose and fructose are the predominant sugars, with variable contents of 18–52% and 14–62%, respectively [36,37,38]. The relative proportions of these sugars vary across studies due to factors such as cultivar, ripening stage, and environmental conditions. This highlights the importance of cultivar selection for specific nutritional or technological applications. Date seeds have a distinct sugar profile to the pulp, containing glucose, fructose, raffinose, stachyose, galactose and sucrose, with total carbohydrate content ranging from 17.4 to 27.8 g per 100 g [39,40].

Although dates are high in sugar, their metabolic impact depends on the context and may vary according to cultivar, ripening stage, portion size and whether they are consumed with fiber- and polyphenol-containing foods. Therefore, health-related statements based solely on sugar composition or in vitro indices should be treated with caution and ideally be supported by controlled human studies.

Dates are widely recognized as a source of dietary fiber, which supports digestive health, lowers cholesterol and reduces the risk of chronic conditions such as colorectal cancer, diverticulosis, appendicitis, varicose veins and hemorrhoids [15]. Their fibers also have functional properties such as emulsification, gel formation and water/oil retention, making them useful ingredients in functional and innovative food products [3,7,18]. 

The dietary fiber content of date pulp typically ranges from 2 to 8 g/100 g [6,41], whereas date seeds contain substantially higher levels, ranging from 10 to 87 g/100 g [42,43]. The fiber fraction is mainly soluble, particularly arabinoxylans and β-glucans, which enhance both nutritional and functional qualities [3,5,18,44]. The technological functionality of date fibers (e.g., water/oil holding and gelation) is highly sensitive to particle size, extraction conditions, and thermal history. Moreover, performance observed in model systems does not always translate to complex food matrices. More standardized characterization (soluble/insoluble fractions and viscosity profiles) would improve comparability across studies and facilitate application-driven selection.

Although dates are not considered a major protein source (1.6–4.7% in pulp; 1.4–4.84% in seed), their protein content is relatively higher than that of many other fruits [5,37,40,43,45]. However, the nutritional significance of these proteins remains limited due to both their low concentration and incomplete amino acid profile. While some studies report appreciable levels of essential and non-essential amino acids, the variability in reported concentrations (0.02–382 mg/100 g) suggests inconsistencies in analytical approaches and cultivar origin [17,46]. Consequently, dates should be viewed as a complementary rather than a primary source of dietary protein. 

Although date pulp contains very low levels of lipids (0.04–0.65%) [38,47,48], it does contain bioactive lipid compounds, such as oxygenated fatty acids and conjugated sphingolipids [15,49]. Date seeds, in contrast, are richer in lipids (7–13%), and their oil contains favorable proportions of saturated and unsaturated fatty acids, including oleic, palmitic, lauric, linoleic, myristic, stearic and linolenic acids [6,50,51]. Variability in lipid composition across studies is often linked to differences in extraction methods, and seed cultivar/origin.

Dates are a notable source of vitamins. The pulp contains vitamin C and B-complex vitamins, including riboflavin, thiamine, pyridoxal and niacin [45,52], as well as vitamins K and A, mainly in the form of β-carotene [5]. Trace amounts of vitamin E are also present [36]. Date seed oil is particularly rich in tocopherols (44.7–110.8 mg/100 g), including the α-, β-, γ- and δ-forms [6,50,53]. However, reported concentrations may vary depending on analytical methods and cultivar.

Dates are often described as mineral-rich fruits. The pulp contains high levels of potassium, sodium, calcium, phosphorus and magnesium [5,20,48,54], while the seeds contain even higher concentrations of minerals, particularly potassium, sulfur, phosphorus and calcium [7,43]. However, reported mineral content varies significantly across studies, reflecting differences in cultivar, soil composition, irrigation practices, and postharvest handling. Notably, trace elements such as Pb and Cd have been detected in some seed samples. This highlights the need for safety-focused monitoring, as mineral profiles may reflect soil contamination and processing conditions. Future food applications should explicitly address contaminant limits and variability between batches to support regulatory acceptance. 

### 3.2. Phytochemicals Content

Phytochemicals comprise a wide array of bioactive compounds that are broadly classified as either primary or secondary metabolites. While this classification is well established, most studies on date fruits focus almost exclusively on secondary metabolites, such as terpenoids, alkaloids, carotenoids, oxylipins, flavonoids and other phenolic compounds, due to their reported biological activities. Date fruits and their by-products are rich in these compounds and regular consumption as part of a balanced diet may help to prevent and manage chronic diseases [7,55]. However, the relative contribution of different phytochemical classes to health effects is not sufficiently compared across studies. This lack of comparative evaluation hinders the identification of the most functionally relevant compounds in dates and their by-products. Moreover, differences in extraction solvents, assay protocols, and expression units across studies contribute to variability in reported results.

Antioxidant activity is often cited as a key factor in the classification of dates as functional foods. Studies on date fruits and seeds have reported strong positive correlations between total phenolic content and antioxidant activity, emphasizing the significant contribution of phenolic compounds to their bioactive properties [56]. Correlations between total phenolic content (TPC) and chemical antioxidant assays are frequently reported. However, these assays reflect reducing capacity under simplified conditions and do not necessarily predict biological efficacy in vivo. Consequently, bioavailability, metabolism, and matrix interactions can substantially modulate the activity of phenolics, which underscores the need to interpret TPC–antioxidant correlations as screening indicators rather than proof of health benefit, which require validation through in vivo and clinical studies. Table 1 summarizes the major phytochemical compounds identified in dates and their associated health-promoting properties.

The phytochemical composition of the date palm can vary considerably depending on genetic factors, cultivar, geographical origin, climatic conditions and agronomic practices, as well as the maturity at harvest and postharvest treatments, such as storage and processing technologies [5,17]. A major limitation of the current literature is methodological heterogeneity in extraction solvents, hydrolysis steps, chromatographic platforms (HPLC-DAD vs. LC–MS/MS), and reporting units, which can lead to inconsistent identification and quantification of individual phenolics across studies. Therefore, harmonized methodologies and the use of reference standards are essential to improve reproducibility, and enable more reliable selection of cultivars/by-products for targeted applications. Table 2 shows the phenolic profiles of different varieties of date fruit and seeds from various geographical locations.

Phenolic compounds are the most abundant and extensively studied class of phytochemicals found in dates. However, reported profiles can vary widely across studies due to differences in cultivar, maturity stage, and analytical approaches. Benzoic acid and cinnamic acid derivatives are consistently identified as major components, along with various related isomers. Flavonoids, generally present at lower concentrations, occur in multiple forms (glycosylated, methylated, or sulfated) and contribute to antioxidant and other biological activities [7,14,34,57]. Carotenoids and proanthocyanidins are typically found at low levels in mature date pulp, but may be more concentrated in seeds. Additional compounds, including sterols, triterpenoids, steroids and phthalates, have also been identified and may contribute to the overall bioactivity of dates [17,58]. However, reports of phthalates should be interpreted cautiously, as they may originate from external contamination (e.g., packaging materials or laboratory plasticware) rather than plant biosynthesis. Whenever detected, confirmation with contamination-controlled protocols is essential before attributing them to intrinsic date phytochemistry. 

Several studies have shown that substantial amounts of bioactive compounds are retained in date processing by-products, supporting their valorization in a circular economy [59,60]. Date seeds, in particular, have been reported to consistently contain higher levels of phenolic compounds, carotenoids and lipophilic substances such as tocopherols and phytosterols [61,62,63]. This compositional enrichment makes date seeds a promising source of functional ingredients [62,64]. However, despite this potential, their industrial application remains limited, partly due to insufficient standardization, safety assessment and techno-functional validation in real food systems. Future research should therefore prioritize in vivo/clinical validation to facilitate the effective utilization of date by-products as functional ingredients.

**Table 1:** Dietary phytochemicals of date palm and their functions and health benefits.

Substance	Common Function and Health Benefits	Reference
*Carbohydrates*		[65,66,67,68]
(Polysaccharides and fibers)	Improved gastrointestinal function (regulate intestinal transit)	
	Inhibition of gluconeogenesis (lowering the plasma glucose response)	
	Protecting against the development of type 2 diabetes (lowering the digestion and absorption of carbohydrates)	
	Reducing the risk of developing obesity and overweight (increasing satiety, decreasing appetite, and controlling energy intake)	
	Anti-inflammation effect	
	Antioxidant and antibacterial activity	
	Treatment of hypoglycemia (provide instant energy for breaking fast)	
	Prebiotic effects	
*Minerals*		[18,54,69,70]
Potassium	Protection against cardiovascular disease (regulate blood pressure and heart rate and maintain balance in the nervous system and body fluids)	
Selenium	Stimulation of immune system	
Iron	Prevention of muscular fatigue	
Zinc	Immune system function	
*Bioactive compounds*		[8,18,71]
Phytosterols	Reduction in LDL cholesterol levels (inhibition of absorption of dietary cholesterol and reabsorption of cholesterol from bile acids)	
	Treating hormone-related health conditions	
	Neuroprotective potential (reduce progression of Alzheimer’s disease and prevention of Parkinson’s disease)	[69,72,73,74]
Ferulic acid	Hinders inflammatory mediators	
Chlorogenic acid	Reduces ROS production by restricting protein carbonylation	
Myricetin	Reduces caspase-3 activation, thereby reducing Aβ plaques aggregation	
Protocatechuic acid	Improves depolarization by activating Na^+^ K^+^ Atpase	
Caffeic acid	Inhibits the synthesis of AChE and the activation of Keap1-Nrf2	
Gallic acid	Decreases the death of dopaminergic neurons	
EGCG	Restricts the aggregation of α-syn protein	
Luteolin	Reduction in oxidative stress	
	Cardio-protective effect (prevention of hypertension, atherosclerosis, myocarditis, and myocardial infarction)	[69,70,75,76]
Quercetin, rutin, and luteolin	Decrease the levels of CRP and homocysteine, and reduce the production of COX-1 and COX-2	
Gallic, ferulic, and hydroxybenzoic acids	Restore the levels of CD34 and CD133 positive progenitor cells and the diminished level of GSH, SOD, and CAT	
	Reduce osteoporosis	[69,77]
Caffeic acid	Prevent oxidative damage to bones by downregulating NOX1 and increasing the expression of osteoblastic-activity genes (BMP-2 and BMP-7)	
Coumarin	Activates IGF-1 and promotes differentiation of bones	
EGCG	Decreases the production of inflammatory mediators and reduces oxidative stress in bones	
Phenolic acids and flavonoids	Anti-atherogenic effect	[54,78,79,80,81,82]
	Antidiarrheal effect	
	Reduced cytotoxicity and aggregation	
Proanthocyanidins	Preventing type-2 diabetes mellitus	
Other compounds	Reduction in hepatorenal toxicity	
	Prevent or treat SARS-CoV-2 infection	
	Reduced blood glucose levels	
	Prevention of various diseases linked to oxidative stress	
	Activation of the immune system	
	Disruption of the bacterial membrane	
	Neutralizes free radicals and prevents cell damage, promotes natural labor and enhances breastfeeding (increasing cervical dilation and uterine contractions, reduce postpartum hemorrhage, and increase milk production)	

ROS: Reactive oxygen species; CD: Cluster of differentiation; AChE: Acetyl cholinesterase; SOD: Superoxide dismutase; GSH: Glutathione; EGCG: Epigallocatechin gallate; NOX: NADPH Oxidase.

**Table 2:** Major polyphenolic compounds from *Phoenix dactylifera* L.

Group of Compounds	Name of Compounds	Content			
		Fruit	Reference	Seed	Reference
*Phenolic acids* (mg/100 g)	Gallic acid Ferulic acid *p*-coumaric Caffeic acid Syringic acid Chlorogenic acid Vanillic acid Protocatechiuc acid Caffeoylshikimic acid Sinapic acid	1.61–16.07 0.26–4.71 0.09–3.27 0.03–1.37 0.55–5.49 0.04–0.30 0.23–2.13 2.27–84.51 - 0.05–1.53	[36,56,83,84,85]	0.24–57.5 2.38–6.93 0.14–11.14 0.11–31.53 0.13–8.43 3.10–8.20 2.95–4.07 0.003–44.81 4.5–28.3 -	[40,50,56,86]
*Flavonoids* (mg/100 g)	Catechin Isoquercetin Rutin Quercetin Quercetrin Luteolin Epicatechin Apigenin Proanthocyanidin Anthocyanin Isorhamnetin Kaempferol Naringenin	0.02–2.98 0.10–3.03 0.17–2.50 0.34–5.81 0.04–1.27 0.02–0.71 0.06–0.66 0.07–1.56 - 0.24–1.52 0.26–9.22 0.04–5.37 0.76–1.19	[36,56,83,84,85]	0.29–171.6 - 0.09–0.12 22.37–107.03 3.29–13.65 - 7–18.8 0.5–41.54 55.8–80.23 0.24–126 41.91 18.65 -	[40,50,56,86]
*Carotenoids* (µg/100 g)	Luteins β-Carotene Neoxanthin Zeaxanthin	45.7–129 3–6.5 184–381 33	[87]	160 314 - 1.08	[63,88]
*Phytosterols* (mg/100 g)	β-Sitosterol Avenasterol Campesterol	- -		228.6–231.5 51.41–53.65 39.25–41.42	[64]
*Tocopherols* (mg/100 g)	α-Tocopherol β-Tocopherol δ-tocopherol γ-Tocopherol	- -		26–54.39 3.20–8.93 4.54–11.31 18.60–51.33	[50,62,64]

## 4. Biofunctionalities of Date and Its Products

### 4.1. Orthodox Medicine and Traditional Therapeutic Application

Dates have long held cultural, nutritional and medicinal value, particularly in Arabic and Islamic traditions, where they are commonly consumed during fasting and religious rituals because of their high energy density and nutritional richness [71]. The date palm (*Phoenix dactylifera* L.) has been used since ancient times for preventive and therapeutic purposes. Different parts of the tree, including fruits, seeds, leaves and sap, have been used in traditional medicine [52,89,90].

Traditionally, date fruits have been used to treat whooping cough, leukoderma and other skin disorders. In Egyptian folk medicine, they are consumed for their hypoglycaemic and antidiabetic effects. In Sudanese traditional medicine, aqueous mesocarp extracts are used against jaundice. Date-based syrups have also been recommended for the treatment of liver disorders and to support women before and after childbirth. Historical records also describe the use of *P. dactylifera* in endocrine and reproductive disorders [52,89,90]. Across North Africa and the Middle East, dates have also been used as a general tonic. In some regions, they are also used to manage diabetes and hypertension [3,91]. Decoctions and infusions have traditionally been used to treat colds, coughs, asthma, chest pain and gastroenteritis. In addition, their laxative properties support their use against constipation and hemorrhoids [91,92]. Due to their high mineral and natural sugar content, dates are considered beneficial for pregnant and breast-feeding women. They are also recommended for individuals with anemia [93,94]. However, these applications are primarily based on traditional knowledge and observational practices rather than standardized clinical evaluation.

**Figure 4:**
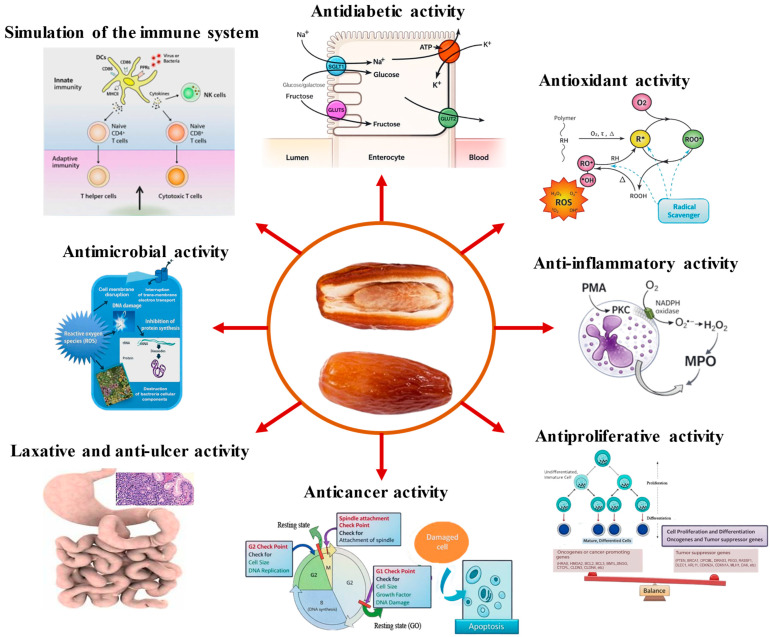
Therapeutic potential applications of date fruit and its products.

Other ethnomedicinal uses include abdominal and intestinal disorders, urinary tract conditions, sore throat, fever, bronchial catarrh, inflammation, gonorrhea, liver edema, alcohol intoxication, paralysis, memory impairment and nervous or mental disorders [90,95,96]. Although these traditional uses are well documented, clinical validation remains limited. Their translation into evidence-based recommendations is further constrained by variability in plant material (cultivar, maturity stage), preparation methods, doses and outcome measures. Nevertheless, recent phytochemical and pharmacological studies have begun to clarify the mechanisms underlying these traditional claims (Table S1; Figure 4). Further standardized and controlled clinical studies are required to confirm efficacy and ensure safe application in modern therapeutic contexts.

### 4.2. Nutraceutical Potential and Health-Promoting Properties

Date fruits and their by-products contain a broad range of bioactive compounds, including phenolic acids, flavo-noids, procyanidins, anthocyanins, carotenoids and phytosterols, which underpin their nutraceutical potential [89,97]. Antioxidant activity is considered one of the main mechanisms behind their health-promoting effects, and may involve synergistic interactions among multiple compounds [6,7,56,71]. Accordingly, date fruits, seeds, and derived products have been associated with multiple bioactivities, including antioxidant, anti-inflammatory, antidiabetic, and antimicrobial effects, among others. These properties may support cardiovascular, gastrointestinal, hepatic, renal, metabolic, and neurocognitive health [5,7,39,69,70,71,89,90,98].

Polyphenols play a central role in these effects. They act by scavenging reactive oxygen species, chelating transition metals and inhibiting pro-oxidant enzymes [90,99,100]. The antioxidant capacity of *P. dactylifera* has therefore been widely assessed using DPPH, FRAP, ABTS and ORAC assays. These assays reflect reducing capacity under simplified conditions and do not necessarily predict biological efficacy in vivo. Furthermore, variability in extraction methods, solvent systems, and assay protocols contributes to discrepancies in reported antioxidant values across studies. Therefore, such results should be interpreted as indicative rather than conclusive evidence of health benefits.

In vivo studies have shown that oral administration of lyophilized date fruit extract preserved endogenous antioxidant systems in rats, including catalase, superoxide dismutase, nitric oxide and non-protein sulfhydryl groups, while significantly reducing lipid peroxidation [101]. Other studies similarly reported increased activities of catalase, superoxide dismutase, gluta-thione reductase, peroxidase and S-transferase, together with lower malondialdehyde levels [76,102,103,104]. However, differences in dosage, extract composition, and experimental design make cross-study comparisons difficult. These factors also limit direct extrapolation to human health outcomes.

Beyond antioxidant effects, date products have shown potential benefits in vascular and cardiovascular health. In vitro and animal studies suggest improvements in plasma lipid profiles, oxidative stress and inflammation markers [70,105]. Their favorable potassium-to-sodium ratio may support blood pressure regulation. In addition, their magnesium and calcium contents contribute to electrolyte balance [106]. In one study, ethanolic date fruit extracts significantly reduced TBARS and troponin-T levels. They also increased reduced glutathione levels, supporting cardioprotective effects [75]. 

Renoprotective effects have also been reported in animal models. Pretreatment with date fruit or seed extracts improved renal morphology and function. It also reduced TNF-α and TGF-β levels, decreased apoptotic markers, and promoted Nrf2 pathway activation [107]. Likewise, in mercuric chloride-induced nephrotoxicity, date extract supplementation significantly reduced plasma urea, creatinine and oxidative stress markers [23]. Nevertheless, these findings are largely based on laboratory assays, and their clinical relevance remains to be established.

Antimicrobial activity against a broad spectrum of pathogenic bacteria has also been documented [108,109,110], probably through membrane disruption, enzyme inhibition and interference with nucleic acid synthesis [106]. In addition, date palm bioactives have shown neuroprotective effects. Long-term dietary supplementation with dates reduced amyloid-β accumulation, ATP depletion, and neuroinflammation in mouse models of Alzheimer’s disease [111]. Other studies reported reduced brain lipid peroxidation and neuronal damage in chronic cerebral hypoperfusion models [112]. These findings suggest possible relevance in neurodegenerative disorders [72,74,113]. However, these effects are highly dependent on extract composition, concentration, and experimental conditions, which vary widely across studies.

Anticancer activity has also been explored. Date seed extracts reduced cancer cell viability and induced apoptosis in breast, liver and colon cancer cell lines [114,115], and in vivo studies showed tumor suppression via modulation of phosphatase and tensin homolog (PTEN) and protein kinase B (Akt) pathways [116]. Moreover, consumption of Ajwa dates during chemotherapy has been associated with reduced risk of infection and treatment-related complications [117,118].

Anti-inflammatory effects have been reported in both human and experimental studies. Date seed consumption reduced pro-inflammatory cytokines and cyclooxygenase expression in human subjects [119], whereas in vitro studies showed inhibition of protein denaturation, stabilization of lysosomal membranes, nitric oxide scavenging, and reduced inflammatory biomarkers [120]. These effects are linked to phenolics such as quercetin, rutin, caffeic acid, p-coumaric acid, and gallic acid.

Date palm bioactives have also been associated with antidiabetic activity. These effects involve inhibition of digestive enzymes, enhancement of glucose uptake, protection of pancreatic cells and reduction in oxidative stress [39,71,106,121]. In vivo studies reported significant reductions in postprandial glycemia and improved glucose metabolism after date extract administration [122,123,124]. Enzyme inhibition studies also demonstrated suppression of pancreatic lipase, tyrosinase and acetylcholinesterase, suggesting additional relevance for metabolic control, skin health and neurocognitive function [40,56,58]. Although still limited, clinical studies provide promising evidence: consumption of date vinegar improved lipid profiles, inflammatory biomarkers and glycemic control in subjects with hypercholesterolaemia and diabetes [125,126,127]. Table S1 (see Supplementary Materials) provides a summary of the therapeutic effects of date palm in the prevention and management of various diseases or health conditions [128,129,130,131,132,133,134,135,136,137,138,139,140,141,142,143,144,145].

Overall, the strength of evidence differs markedly among claimed bioactivities. Most mechanistic data derive from in vitro assays and animal models, often using extracts and doses that may not reflect realistic dietary exposure. In contrast, human studies remain limited in number, sample size, and duration. In addition, cultivar variability, processing conditions and the lack of standardized phytochemical profiling hinder direct comparison across studies. Therefore, while date fruits and their by-products exhibit promising nutraceutical potential, specific therapeutic claims should still be considered preliminary and require validation through adequately powered and well-controlled clinical trials.

## 5. Development of Value-Added Foods from Date Fruits and Their By-Products

Dates are widely valued for their sweetness and functional properties. Dates and date processing by-products offer opportunities for developing value-added products and functional ingredients. Interest in this area has intensified in recent years due to increasing global demand for sustainable food production and circular bioeconomy approaches, as well as for healthier ingredient alternatives [14]. Studies report promising nutritional and technological improvements following the incorporation of date-based ingredients. However, most research remains at laboratory or pilot scale. Few studies address industrial scalability, cost–benefit analysis, regulatory constraints or long-term consumer acceptance. Therefore, while the potential for valorization is substantial, translation into commercially viable products requires more systematic techno-economic and sensory validation.

Despite being perceived as overly sweet or high in calories, dates offer significant nutritional and functional benefits. Expanding their industrial use beyond local markets could diversify products and promote the incorporation of date-derived ingredients into various foods. However, this requires closer collaboration between researchers and industry. Such collaboration is needed to optimize processing, ensure quality and safety, and develop products that are both acceptable to consumers and supported by proven health benefits [1].

(a) Natural sweeteners: the high intake of refined sugars has been linked to adverse health effects, which has increased interest in natural sweeteners. Date fruit is a promising alternative, providing natural sweetness as well as minerals and phytochemicals that can improve the nutritional value of food [146].
  - Date syrup is widely used in baking, beverages and fermentation products. It is often produced from overripe dates. Its production involves multiple processing steps, and using enzymes to assist the extraction process improves yield and quality [146,147]. Studies show that using whole dates improves the nutritional and sensory properties [148], while extraction methods affect the yield and composition, and evaporation mainly influences the color [149].
  - High fructose date sugar (liquid): is a concentrated sugar solution (70–80% total solids), typically produced by extracting date juice and concentrating it under vacuum. It is widely used in jams, beverages, baked goods, and confectionery [123].
  - Date sugar powder is made from dehydrated dates or low-moisture date paste that has been milled into a powder. It has a caramel-like flavor and is commonly used as a natural sweetener in dry mixes and bakery products [25,150].
  - Date juice and concentrates are often produced from low-value dates through grinding, heating, pressing, and clarification steps [1,151]. Advanced processing methods, such as thin-film evaporation, can improve quality. The increasing popularity of date-based drinks highlights their strong market potential [150,151].
  - Date jam and jelly: date jam is made from ripe date pulp, which is mixed with water, sugar and pectin. The pH is adjusted to between 3.0 and 3.5 and the mixture is concentrated to between 60 and 70 °Brix. Date jelly is made in a similar way, using pulp or juice and sugar. It is concentrated to around 65 °Brix and preservatives are added if necessary [150].
  - Date paste is a semi-solid product used in baking and confectionery, as well as in powders, syrups and candies. It is made by macerating or steaming pitted dates, to which antioxidants and acidulants are added to enhance shelf life, color and texture stability [150].

Date-derived sweeteners are often promoted as a healthier alternative to refined sugars. However, their high intrinsic sugar content requires careful nutritional positioning. Their metabolic impact depends on portion size, food matrix and degree of processing. Current evidence does not consistently demonstrate superiority over conventional sugars in terms of glycemic control in controlled human trials. Furthermore, compositional variability between cultivars and processing methods may affect standardization and labeling consistency.

(b) Fermented products: fermentation is a promising approach for converting dates and their by-products into higher-value commodities, given their high carbohydrate content [35]. Enzyme-assisted and fermentation processes have been investigated for transforming date waste into biofuels, biopolymers and industrial enzymes [152]. Date substrates have been investigated for use in producing:
  - Vinegar and Alcohol: the production of alcohol and vinegar from dates involves fermentation processes. Ethanol is produced by fermenting sugars (typically using *Saccharomyces* spp.), and date seeds have been investigated as a lignocellulosic source of bioethanol via cellulose extraction, enzymatic hydrolysis and fermentation [153,154]. Date vinegar is produced through a process of sequential alcoholic and acetic fermentation, resulting in products that contain higher levels of phenolic and carotenoid compounds, and exhibit stronger antioxidant activity than certain commercial vinegars [150,155,156].
  - Organic acids: date syrups and processing waste can be used to produce organic acids. Fungal fermentation of date syrup can be used to produce citric acid, while enzymatic saccharification followed by bacterial fermentation can be used to produce lactic acid from date waste [152,157]. Studies show that lactic acid can be produced from date press cake using *Lactobacillus casei* [158], and that the production of citric acid can be enhanced using date pit hydrolysate supplemented with pit ash [159].
  - Microbial biomass/starter cultures (probiotics/yeast production): using date juice and syrup provides an eco-friendly, sustainable carbon source for producing baker’s yeast. *Saccharomyces cerevisiae* is an effective yeast for producing baking yeast from these substrates [152].

The fermentation of date by-products is conceptually aligned with the principles of the circular bioeconomy. However, comparative life-cycle assessments and industrial feasibility studies remain limited. The environmental and economic sustainability of these processes depends on factors such as energy input, enzymatic costs, and downstream purification requirements.

(c) Flours and powders: dried dates can be processed into gluten-free flours and powders for use in baking. Production methods include pitting, cutting, dehydrating, milling and sieving, or spray drying with carriers such as maltodextrin and gum Arabic, which enhance stability and flow [25]. The resulting powder can be used as a natural sweetener and a formulation aid [160].
(d) Date fiber concentrates: the skin and pulp of dates are rich in dietary fiber and can be used in supplements and functional foods. Fiber concentrates offer nutritional and technological benefits such as water/oil absorption and thickening. Processing methods, including hot-water extraction and enzymatic treatment, can increase the content of soluble fiber, antioxidants and functional oligosaccharides. This supports the development of prebiotic ingredients [44,161].
(e) Biomass utilization: date processing by-products can be valorized through microbial fermentation to produce biomass and synthesize compounds such as oxytetracycline and gamma-aminobutyric acid. Common microorganisms used for this process include *lactobacilli*, *Streptococcus thermophilus*, *Streptomyces rimosus*, *Corynebacterium glutamicum* and yeasts [152].
(f) Date seeds as food ingredients and seed oils: date seeds can be used as flour in bakery products, roasted as a caffeine-free coffee substitute or processed into seed-based beverages. Date seed powder is commercially available and promoted as being nutrient-dense. Date seed oil is rich in oleic–lauric lipids and shows high oxidative stability. This suggests its suitability for use in cooking oils, margarines and mayonnaises [51,150,162]. Additionally, date seeds can be processed into fiber and protein concentrates for food applications. The resulting fiber concentrates are rich in dietary fiber (~50–70%), cellulose, and hemicellulose. They can be used to create fiber-enriched foods and provide functional properties such as emulsification [163,164]. Ultrasound-assisted processing has been explored to enhance the solubility and technological performance of seed protein concentrates [165].

The wider use of date by-products in food requires robust standardization, quality control, and safety assessment. In addition, processing conditions must be optimized to ensure consistent consumer acceptance and regulatory compliance. Despite the promising oxidative stability and bioactive profile of date seed oil, regulatory approval, allergenicity assessment and long-term safety evaluation remain inadequately addressed in the literature. Additionally, variability in lipid composition across cultivars and extraction methods may complicate standardization for large-scale food application.

## 6. Potential Industrial Application of Date Fruits and Their By-Products

**Figure 5:**
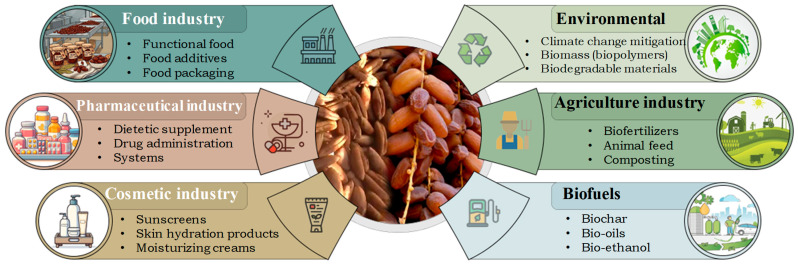
Potential industrial application of date fruit and its seed.

Dates and their processing by-products are increasingly recognized as promising raw materials for industrial applications, particularly in functional foods. This is due to their content of fiber, minerals, proteins, polyphenols and other bioactive compounds. Beyond their traditional consumption as fresh or dried fruits, dates are processed into juices, syrups, pastes, sugars and jams. This generates by-products such as seeds and pomace that can be further valorized [8]. This valorization contributes to waste reduction, resource efficiency and the recovery of high-value ingredients for food, cosmetic and pharmaceutical applications (Figure 5). However, despite the frequent association of these strategies with sustainability, comparative data on carbon footprint, water use and energy demand remain limited. Therefore, environmental benefits are still insufficiently quantified [166].

### 6.1. Food Industry

Growing consumer demand for healthier foods has stimulated the incorporation of date fruits and their by-products into value-added formulations. Date paste, syrup and seed derivatives have been used in bakery, dairy, meat, beverage and confectionery products as natural sweeteners and functional ingredients. In many cases, they improve nutritional value, antioxidant capacity, and shelf life while maintaining acceptable sensory quality [1,54,166,167]. The main applications are summarized below and in Table S2.

- Bakery products

Bakery products are among the most studied matrices for date-based ingredients. Date derivatives, including powder, paste, syrup, pomace and seed flour, have been incorporated into bread, biscuits, cakes, muffins and pasta. In general, their incorporation increases fiber, mineral and phenolic contents and, in some cases, improves antioxidant activity and storage stability [8,18,167]. For example, partial replacement of sucrose with date pulp in bread increased fiber and protein levels [168], while date seed powder enhanced the phenolic content and antioxidant activity of pita bread and reduced acrylamide formation at 5% addition [169]. Similarly, date press cake powder improved the nutritional value and sensory acceptance of biscuits and protein bars. It also maintained microbiological stability during storage [170]. Date syrup has also been used successfully as a sugar substitute in sponge cake, increasing total phenolics and antioxidant capacity without markedly compromising texture [171].

Overall, date-derived ingredients show clear potential in bakery reformulation, although high inclusion levels may negatively affect dough rheology, loaf volume and crumb structure, and many studies remain limited to laboratory-scale conditions.

- Dairy products

Date fruits and by-products have also been incorporated into dairy desserts, cheeses, yogurts and fermented milks to improve nutritional and functional quality. The replacement of added sugar with date syrup or powder has been associated with increased antioxidant capacity and polyphenol content [172], whereas date seed fiber has been tested as a partial butter substitute in processed cheese, yielding acceptable physicochemical and sensory properties at moderate inclusion levels [173]. In probiotic and fermented dairy products, date juice, syrup, paste or flour have been shown to enhance mineral content and antioxidant activity. In some cases, they also improve probiotic viability and sensory acceptance [173,174,175,176,177].

Despite these promising results, phenolic stability during storage is variable, and excessive fortification may adversely affect texture, firmness or syneresis, which could limit industrial application unless formulations are optimized.

- Meat products

Date-derived ingredients have been explored in meat products as extenders, fat replacers and natural antioxidants. Their incorporation may improve water-holding capacity, oxidative stability, cooking yield and nutritional value by increasing fiber and reducing fat content [1,167,178]. Date pomace fiber has shown technological potential in plant-based meat analogs [179], while date seed powder reduced lipid oxidation, delayed microbial growth and improved cooking performance in beef burgers [180]. Likewise, soluble and insoluble fibers isolated from date seeds have been used successfully in burger reformulation. In most cases, moderate inclusion levels provide the best sensory and technological outcomes [181,182].

However, excessive fiber incorporation may impair texture, juiciness and consumer acceptance, and broader validation across products and markets is still required.

- Beverages

Date seed powder has attracted attention as a caffeine-free alternative in coffee-like beverages. Roasted date seed drinks show acceptable sensory similarity to conventional or Arabic coffee, although their sensory scores are usually lower in direct comparisons [183,184]. Roasting conditions strongly influence the chemical composition and organoleptic quality of the final beverage. Optimized roasting can improve overall acceptability [185,186]. More recent strategies, such as blending date seed powder with Arabica coffee or applying freeze-drying, have further improved aroma retention, solubility and antioxidant activity [187]. 

These beverages are promising functional products. However, their market potential may be greater in niche health-oriented segments than as direct substitutes for conventional coffee.

- Confectionery products

Date-based ingredients are increasingly used in functional confectionery products such as snack bars, jams, sweets and chocolate sauces. Date paste is widely employed in protein bars as a natural sweetener and binder, often in combination with protein-rich ingredients, resulting in products with improved fiber content, antioxidant capacity and nutritional quality [6,188]. In addition, date press cake and date seed powder have been successfully incorporated into protein bars, chocolate sauces and jams, improving nutritional composition, sensory acceptance and storage stability [170,182,189]. These findings support the use of date by-products as functional and cost-effective ingredients in confectionery applications.

- Food Packaging

Date fruits and especially date seeds have also shown potential in active and biodegradable food packaging systems. Their incorporation into films and coatings can provide antioxidant and antimicrobial properties. It may also improve certain structural and barrier characteristics [190]. Date seed powder has been incorporated into starch- and cellulose-based composite films [191,192]. Date seed oil has been applied as an edible coating to prolong guava storage [193]. In addition, extracts derived from date syrup waste or date fruit have improved the antioxidant and antimicrobial performance of gelatin- and chitosan-based films [194,195]. In some cases, these materials extended the shelf life of fruits such as strawberries and blueberries [195,196].

Nevertheless, further research is needed on migration behavior, mechanical resistance under real distribution conditions and regulatory compliance for food-contact applications. In addition, increased bioactivity may compromise film strength unless composite strategies are used [197].

Additional studies investigating the use of date-derived materials in food applications are summarized in Table S2 (see Supplementary Materials) [198,199,200,201,202,203,204,205,206,207,208,209,210,211,212,213,214,215,216,217,218,219,220,221,222,223,224,225,226,227,228,229,230,231,232,233,234,235,236,237,238,239,240,241,242,243,244,245,246,247,248,249,250,251,252,253,254,255].

### 6.2. Cosmetic and Pharmaceutical Industry

Growing consumer demand for plant-based alternatives has led to increased interest in natural, renewable bioactive compounds within the cosmetics and pharmaceutical industries. Date fruit extracts and seed oil are rich in antioxidants, vitamins, fatty acids, oxylipins, and other phytochemicals. They have therefore emerged as promising ingredients for these applications. Research highlights their potential in skincare, showing benefits for hydration, elasticity, pigmentation, and protection against oxidative stress [256,257]. However, most studies are limited to small-scale trials, and long-term safety, allergenicity, and regulatory approval remain inadequately addressed.

Several studies have highlighted the cosmetic potential of date-derived ingredients. Topical creams containing date fruit extract or seed oil have been shown to improve skin elasticity, hydration, pigmentation, and overall appearance. They may also replace synthetic components with natural alternatives [258,259]. Date extracts contain vitamins such as C and E. They may enhance skin structure and resilience by stimulating dermal fibroblast activity and collagen synthesis [105]. Additionally, the use of antioxidant-rich date syrup biomass has been explored as a sustainable substitute for synthetic chemicals in soap production, offering further industrial applications [260].

With its beneficial fatty acid composition and polyphenol content, date seed oil shows promise as a natural excipient in topical formulations and hair care products. Its UV absorption and antioxidant properties suggest potential applications in anti-photoaging products [49]. Additionally, its omega-3 content may promote hair health. Reported effects include improved scalp condition, stimulated growth, reduced dandruff, and stronger hair fibers [154,256]. However, variability in oil composition across cultivars and extraction methods complicates standardization for commercial use.

Interest in date seed oil for pharmaceutical and nutraceutical applications is growing due to its antioxidant properties, particularly when it is obtained through eco-friendly extraction methods. Its triterpenoid and steroid content offer adaptogenic and anabolic benefits. In addition, its fatty acid profile, which is rich in stearic, palmitic, and oleic acids, makes it a suitable functional excipient. The oil has been suggested as an adjuvant in topical anti-inflammatory formulations, improving the absorption of non-steroidal anti-inflammatory drugs rather than acting as the primary active ingredient [51]. 

Incorporating date-derived ingredients into cosmetic and pharmaceutical products offers a sustainable, multifunctional approach. This trend is aligned with the growing demand for natural, bio-based, and health-focused formulations. Future research should focus on standardized extraction, robust clinical validation, regulatory assessment, and industrial scalability to enable consistent and safe product development.

### 6.3. Agricultural and Environmental Application

Date processing by-products are abundant, low-cost and rich in organic matter. They are therefore increasingly being studied for use in agriculture and environmental applications. These residues can be converted into high-quality organic fertilizers through composting via windrow, vermicomposting, or in-vessel methods. These fertilizers improve soil structure, nutrient content, microbial activity, and plant growth. They therefore support sustainable agriculture [154,166]. 

Date seeds have also been investigated as a low-cost, environmentally friendly adsorbent and as a precursor for the production of biochar and activated carbon. The porous structure, high surface area, and functional groups of date seed biochar enable effective adsorption of a wide range of contaminants from aqueous systems. These contaminants include heavy metals, dyes, pesticides, and phenolic compounds [261,262].

Date seed-derived biochar has several advanced applications. One example is its use as a KOH-activated electrode material in capacitive deionization for desalination processes. Its porosity and electrochemical performance can be adjusted by varying the amount of KOH used [263]. Date seed-derived activated carbon also exhibits strong adsorption capacity for contaminant removal, highlighting its potential in wastewater treatment and environmental remediation [264].

Date processing by-products can be used to produce animal feed, providing a sustainable circular bioeconomy solution. Rich in fiber, protein, fat, and carbohydrates, they support the nutritional needs of livestock, poultry, and aquatic species. Date pit residues obtained after oil extraction have been shown to increase protein and triglyceride levels in lactating animals [51,154]. In addition, palm date meal improves the growth and health of fish and may reduce the need for antibiotics [265]. 

Despite these promising results, most applications have only been demonstrated at laboratory or pilot scale. Comparative life-cycle assessments, techno-economic evaluations, and scalability studies are needed to confirm the environmental benefits and facilitate industrial adoption.

### 6.4. Biofuel Application

Date processing by-products, particularly the seeds, are a promising renewable resource for sustainable energy. Due to their high oil content and widespread availability, date seeds have emerged as an attractive feedstock for biodiesel production. They yield a biodegradable fuel with lower emissions than fossil fuels [51,154,266]. Their low free fatty acid content enables cost-effective transesterification. Catalysts such as NaOH, KOH, or eco-friendly, waste-derived options like eggshells can be used. This produces biodiesel that meets international fuel standards [267,268,269].

Date seed biomass is a versatile source of sustainable energy. In addition to biodiesel production, date seed biomass can be converted via pyrolysis into stable oils. These oils can then be hydro-processed into deoxygenated hydrocarbons for use as fuels [166,270]. Additionally, its high fermentable sugar content enables the production of biological energy, including biogas. Studies have demonstrated the successful anaerobic digestion of residual date fibers for biogas generation [271,272,273].

Date seed by-products, which are rich in lignocellulosic biomass, are a promising substrate for biohydrogen production [157]. Microorganisms such as *Clostridium thermocellum* convert the reducing sugars in these by-products into hydrogen. This process also generates acetate, butyrate, and ethanol as by-products. Process efficiency can be improved using surfactants [154,274]. Integrated approaches combining dark fermentation of date flesh and anaerobic digestion of residual fibers have also been shown to maximize biohydrogen and methane yields [275].

Overall, the use of date fruits and their processing by-products for biofuel production provides a sustainable approach to renewable energy and waste valorization. It also supports the development of a circular bioeconomy. However, continued optimization of conversion technologies and process integration is crucial to improving economic feasibility and enabling industrial-scale implementation.

## 7. Limitations and Challenges

Importantly, several cross-cutting limitations constrain both scientific interpretation and industrial translation. First, substantial variability across cultivars, geographical origins, ripening stages, and processing conditions limits comparability between studies. This problem is compounded by heterogeneous extraction protocols and analytical techniques, which complicate standardization for formulation and labeling. Second, many reported bioactivities derive from in vitro assays and animal models using extracts and doses that may not reflect realistic dietary exposure. In contrast, human intervention studies remain limited in number, sample size, duration, and product characterization. Third, the stability, bioaccessibility, and bioavailability of key phytochemicals during food processing, storage, and digestion are not consistently assessed. This limits the ability to link compositional richness to clinically meaningful outcomes. Finally, from an industrial perspective, robust techno-economic evaluations, life cycle assessments, and regulatory-oriented safety studies remain scarce. This includes studies on contaminant monitoring and food contact compliance for packaging applications. Addressing these gaps will be essential to support credible health claims and enable large-scale valorization within circular bioeconomy frameworks. This will require standardized methodologies, well-designed clinical trials, and application-driven validation. 

## 8. Conclusions

This review highlights the wide diversity of bioactive compounds present in date fruits and seeds. It also emphasizes their significant potential as ingredients for functional foods and health-oriented products. The available evidence indicates that date-derived bioactives, including dietary fiber, polyphenols, minerals and other phytochemicals, may contribute to beneficial biological effects. These effects are mainly related to metabolic health, oxidative stress modulation and gut microbiota balance. At the same time, numerous technological studies demonstrate that dates and their by-products can be successfully incorporated into a broad range of food formulations. Their incorporation may improve nutritional quality and biofunctional properties while supporting the valorization of agro-industrial residues.

Despite these promising findings, several important research gaps remain. Much of the current knowledge is based on in vitro assays and animal models, while well-designed human intervention studies are still limited. Existing clinical trials are often constrained by small sample sizes, short durations and heterogeneous experimental designs. In addition, differences in cultivars, extraction methods, and processing conditions complicate the comparison of results and the standardization of functional ingredients.

Future research should therefore prioritize well-controlled randomized clinical trials using realistic food matrices. These studies are needed to confirm the bioavailability, efficacy, and safety of date-derived bioactive compounds. Further studies are also needed to clarify dose–response relationships, interactions with the food matrix and the gut microbiome. They should also examine the stability of key phytochemicals during processing and digestion. From a technological and industrial perspective, techno-economic analyses, scalability studies, and process optimization will be essential to facilitate the transition from laboratory-scale demonstrations to commercial applications.

Overall, date fruits and their by-products represent a valuable multifunctional resource for the development of functional foods and other bio-based products. Their efficient valorization could contribute to reducing agro-industrial waste, strengthening circular bioeconomy strategies, and supporting sustainable food systems. With increasing consumer demand for natural and health-promoting ingredients, date-derived products offer a promising platform for future innovation across multiple sectors.

## Data Availability

No new data were created or analyzed in this study. Data sharing is not applicable to this article.

## Supplementary Materials

The following supporting information can be downloaded at: https://www.mdpi.com/article/10.3390/molecules31071194/s1, Table S1: Therapeutic effects of date palm in the prevention and management of various diseases or health conditions; Table S2: Summary of different studies about the industrial food applications of date palm and its by-products. References [128,129,130,131,132,133,134,135,136,137,138,139,140,141,142,143,144,145] and [198,199,200,201,202,203,204,205,206,207,208,209,210,211,212,213,214,215,216,217,218,219,220,221,222,223,224,225,226,227,228,229,230,231,232,233,234,235,236,237,238,239,240,241,242,243,244,245,246,247,248,249,250,251,252,253,254,255] have been cited to Supplementary Materials or in main text.
