# Supplementary material for: Valorization of Date Palm (Phoenix dactylifera L.) Fruits and By-Products as High-Value Sustainable Products: A Comprehensive Review on Bioactive Composition, Health Benefits, and Industrial Applications
Source: Molecules. 2026 Apr 3;31(7):1194. doi: 10.3390/molecules31071194 (PMC13074339; doi:10.3390/molecules31071194)
Supplement: Supplementary file 1 [file molecules-31-01194-s001.zip › molecules-4190333-supplementary.pdf]

Table S1. Therapeutic effects of date palm in the prevention and management of various diseases or health conditions.

Functionality	Dosage or Concentration	Intervention duration	Tested Systems	Result/ Main Outcome	Reference
Nephroprotective Effect (in vivo)	4 and 5 mg/kg body weight (fruit extract)<br>10 and 5 mg/kg body weight (seed extract)<br>(methanolic and aqueous extracts)	-	Male rats	- Improved kidney function and morphology.<br>- Increased expression of Nrf2, catalase activity, and gluthation concentration.<br>- Reduced serum TNF-α and expression of caspase-3, and cytokines (TNF- α and TGF-β).	[107]
	500 and 1000 mg/kg of n-butanol fraction of date fruit extracts	2 weeks	Male Wistar rats	- Restored the changes induced by oxidative stress (alteration the levels of urea, creatinine, and GPx) in the rats exposed to $HgCl_2$.<br>- Protect the kidney against mercury-induced toxicity.	[23]
hypolipidemic and hepatoprotective (in vivo)	1000 mg/kg body weight (seed extract)	30 days	Male Wistar rats	- Lowered the serum cholesterol level and reduced the levels of ALT and AST, demonstrating the hepatoprotective effect in combination with the toxicity caused by the atorvastatin.	[102]
Antihyperlipidemic (in vivo)	25, 50, and 100 mg/kg body weight (fruit extract)	28 days	Male Wister Albino rats	- Improved lipid profiles and antioxidant levels in the plasma and liver tissue.<br>- Reduced total cholesterol, low-density lipoprotein cholesterol, very-low-density lipoprotein cholesterol, and triglyceride levels.<br>- Increased the high-density lipoprotein cholesterol levels, thereby improving the atherogenic index	[128]
Hepatoprotective (in vivo)	1 g/kg body weight (aqueous seed extract)	4 weeks	Adult male Wistar rats	- Attenuate the mitigation of DNA damage and decrease lipid peroxidation and fibrotic changes in liver, as well as normalizing serum levels of hepatic markers (AST, ALT, ALP and albumin).<br>- Restored the activities of hepatic antioxidant enzymes (SOD and GST).	[129]
	100 mg/kg body weight (aqueous fruit extract)	16 weeks	Male Wistar rats	- Increased body weight, improved hepatic architecture, and reduced collagen fiber accumulation.	[130]

				- Reduced liver enzymes, hydroxyproline, alpha-fetoprotein (AFP), malondialdehyde (MDA), inflammatory cytokines (TNF-, NF-B) levels, and DNA fragmentation, while increasing deteriorated adiponectin (ADP) and antioxidant enzymes (GSH, GPX, NO, and IFN) levels.<br>- Suppressed hepatic fibrosis and improved liver function and structure by modulating oxidative stress, inflammation, and apoptosis.	
In vitro	15, 30, and 60 µg/mL of ethanolic date fruit extracts	24 H	Human hepatoma (HepG2) cells	- Increased cell viability, reduced LDH leakage, restored cellular morphology, and inhibited caspase-3 enzyme activity.<br>- Restored the LPO, GSH, and catalase levels.<br>- Significant inhibition of ethanol-induced ROS generation.<br>- Normalized mRNA expression of genes related to apoptosis (p53, caspase-3, caspase-7, Bax, and Bcl-2).	[131]
Anti-tumor (in vitro)	10 to 100 µg/mL of aqueous date seed extract	24 H	Cancer cell lines (human colorectal cell line (HT-29), and human lung cell line (A549))	- The extracts showed dose-dependent anti-tumor activity in both cell types.<br>- The extracts showed higher effectiveness in reducing cell viability in HT-29 than in A549.	[110]
Anticancer (in vitro)	10 to 100 µg/mL of date seed extract		Human cancer cell lines (human triple-negative breast cancer cell line MDA-MB-231, human estrogen receptor and progesterone receptor-positive breast cancer cell line MCF-7, human liver carcinoma cell line HepG2, and normal kidney epithelial Vero cell lines)	- Induced apoptosis in both breast and liver cancer cells through intrinsic pathways.<br>- Treated cancer cells displayed morphological changes after 24 h of exposure.<br>- No changes in morphology or cell viability were observed in the normal Vero cell line following 24 h or 48 h of exposure.	[115]

	50 and 3,000 µg/ml (seed extract)	72H	Human cancer cell lines (breast adenocarcinoma cell lines MCF-7 and MDA-MB-231, colon adenocarcinoma cell line Caco-2, hepatocyte carcinoma cell line HepG2, and human prostate adenocarcinoma cell line PC-3)	- Reduced viability of cancer cell lines (at 1000 µg/mL or above) after 48 h of treatment for MDA-MB-231, MCF-7, and Caco-2 cells and after 24 h for HepG2 and PC-3 cells.	[114]
	0.5, 1, and 2.5 mg/ml (seed extract)	72 H	Human triple negative breasts cancer tissues derived cell line MDA-MB-231	- Seed extracts have cytotoxic effects on MDA-MB-231 cells and altering their morphology aggressively.<br>- Affected the expression of 17/43 kinases at different levels including EGFR, eNOS, ERK1/2, FAK, Fgr, Fyn, GSK3 alpha/beta, Lck, p53, p70S6, PRAS40 and YES.	[132]
	100 to 600 µg/mL (methanolic date seed extracts)	24 H and 48 H	MTT Test with HSF, Caco-2, HepG-2, and MDA cell lines	- Downregulated oncogenes (BCl-2 and P21) and upregulated the pro-apoptotic gene (P53) in the treated cell lines.<br>- Suppressed the expression of both BCl-2 and P21 genes, and increased the expression level of P53 compared to untreated cells.<br>- Exhibited an apoptotic effect on HepG-2 cells.	[133]
Anti-inflammatory (in vivo)	30 mg/kg body weight (seed extract)	-	Wistar strain rats and Swiss albino mice	- Inhibited protein denaturation, stabilized lysosomal membranes; they also demonstrated ability to scavenge nitric oxide free radicals, and inhibit C-reactive protein and fibrinogen production.	[120]
	50 mg/kg body weight (fruit extract)	5 H	Adult male Albino's mice	- Reduced the edema size and the levels of the homocysteine and C-reactive protein in the blood.	[134]
Angiogenic and anti-inflammatory (in vitro)	60 and 600 µg/mL (date syrup extracts)	24 H	Human Vascular Endothelial Cell line (HECV)	- Reduced the expression of vascular endothelial growth factor and the prostaglandin enzyme cyclooxygenase-2 in HECV.<br>- Inhibited TNF-α-mediated endothelial invasion, migration, MMP-2 activity and tube formation.	[135]

				- Reduced the pro-inflammatory cytokines IL-8 and IL-6	
Cardioprotective, anti-apoptotic, and anti-inflammatory (ex vivo)	250 and 500 mg/kg. body weight 250 µl/mL (aqueous fruit extract)	21 days	-Albino male Wistar rats; -Human cardiomyoblast cells (H9C2)	- Reduced cytotoxicity and increased H9C2 proliferation by up to 40%. - Prevented the depletion of endogenous antioxidants (CAT, SOD, NP-SH and NO) and inhibited myocyte injury marker enzymes, and prevented lipid peroxidation (MDA, MPO). - Downregulated the expression of proinflammatory cytokines (IL-6,IL 10 and TNFα) and apoptotic markers (caspase 3 and Bax), and upregulated the anti-apoptotic protein Bcl2. - Reduced myonecrosis, oedema, and infiltration of inflammatory cells and restored the cardiomyocytes architecture.	[101]
Cardioprotective (in vivo)	200 and 400 mg/kg body weight (date fruit ethanolic extracts)	28 days	Male Wistar rats	- Improved the state of myocardial infarction. - Increased the levels of GSH, SOD, and CAT, and decreased the levels of TBRS. - Reduced necrosis, edema, and restored the cardiomyocyte architecture and preserved cardiac muscle fibre morphology. - Increased the reserves of CD34- and CD133- positive cells in the bone marrow.	[75]
Antidiabetic (in vivo)	1g /kg body weight daily (aqueous seed extract)	4 weeks	Adult male Wistar rats	- Protected against early diabetic complications in the liver and kidneys. - Reduced blood glucose levels by 51%. - Decreased total cholesterol, triglycerides, serum urea, and creatinine levels. - Restored AST and ALT levels to normal.	[103]
In vitro	40 and 100 µg/ml (ethanolic seed extract)	-	HepG2 liver cells culture	- Inhibited digestive enzymes responsible for starch digestion glucose uptake modulation by HepG2 cells. - Stimulated glucose uptake by HepG2 cells. - Increased expressions of P-AMPK and AMPK proteins.	[124]
Antihyperglycaemic (in vivo)	200 mg /kg body weight (ethanolic fruit extract)	90 min	Male albino mice	- Decreased plasma glucose levels after 30 min, more effectively than the Acarbose.	[122]
Antidiabetic	10.31 to 13.54 µg/mL (acetonic	-	The inhibits α-glucosidase of	- Inhibited α-GLU in a dose-dependent manner, with 95% inhibition at 25 µg/mL.	[40]

(in vitro)	seed extracts)		*Saccharomyces cerevisiae* was assessed in 96-well microplates	- Exhibited greater inhibition than acarbose.	
Anti-inflammatory and anti-rheumatic (in vivo)	30 mg/kg (n-Hexane and 70% aqueous methanolic seed extras)	4 weeks	adult male Wistar albino rats	- Reduced paw swelling, edema, and neutrophil infiltration.<br>- Reduced the levels of NF-κB, TNF-α, Interleukins 6, 17, 22, 23, 36, and 1β, IFN, JAK1, and STAT3.<br>- Reduced the degenerative alterations caused by rheumatoid arthritis.	[24]
Anti-arthritic effect (in vivo)	500 mg/kg body weight (methanolic fruit extracts)	30 days	Adult female albino Wistar rats	- Maintained homeostasis in the entire metabolism; reduced arthritis symptoms and strengthened the bones.<br>- Normalized the levels of urea, uric acid and creatinine.<br>- Suppressed the development of adjuvant arthritis by inhibiting the chronic phase of inflammation.	[77]
Neuroprotective effect (in vivo)	500 and 1000 mg/kg body weight (aqueous fruit extracts)	28 days	Adult male Wistar rats	- Decreased MDA levels and increased SOD and GSH activities.<br>- Observed mild distortion to relatively normal neuronal cytoarchitecture.	[74]
	2% and 4% date fruit diet	14 months	Transgenic mice	- Lowered the risk of AD and delayed its onset and progression.<br>- Lowered the levels of both Aβ proteins.<br>- Improved cognitive flexibility with effects on anxiety-related behavior, spatial learning, position discrimination, and motor coordination.	[72]
Anti-proliferative (in vitro)	500 μg/mL (date fruit n-butanol extract)	72 H	Human pancreatic cancer cell lines: PT45P1, PANC-1, and SUIT-2	- Significant inhibition of cell viability.	[136]
Radioprotective (in vivo)	4 ml/kg body weight of date syrup / day	4 weeks	Male Wistar albino rats	- Pretreatment with date syrup improved liver function and antioxidant status, reducing tissue and DNA damage.<br>- Reduced liver TNF-α expression and serum MMP-9 activity.	[137]
Improve inflammation, oxidative stress, and cardiometabolic status	5g /day (date seed powder)	8 weeks	Diabetic participants (n = 46)	- Decreased the total cholesterol, triglyceride, low density lipoprotein cholesterol, glycated hemoglobin A1c, lipopolysaccharide, tumor necrosis factor A, oxidative stress index, malondialdehyde, 8-hydroxy-2′-deoxyguanosine, and oxidized-LDL-c	[104]

(triple-blinded randomized placebo-controlled trial)				- Increased total antioxidant capacity.	
Reproductive health effect (randomized controlled clinical trial)	7 date fruits (80 g)/ day	Not reported	Nulliparous singleton pregnant women (n = 154)	- Increased interval of intervention to delivery.<br>- Reduced need augmentation in intervention groups.<br>- Shortened latent, the first and the third phases of labor.	[138]
	7 dates with 250 mL water or without water	During the active phase of labor	Pregnant women (n = 89)	- Significant positive impact on maternal outcomes during the first and third stages of labor.<br>- Higher 5-minute Apgar scores among infants whose mothers consumed date fruits.	[139]
Antidiabetic (type 2 diabetes) (randomized controlled trial)	3 dates/day	16 weeks	Diabetic participants (n = 100)	- Decreased total cholesterol and increased high-density lipoprotein.<br>- Improved the overall quality of life of diabetic individuals.<br>- No increase in triglyceride and low-density lipoprotein levels.<br>- No effect on body mass index.	[140]
Antidiabetic (type 2 diabetes) (randomized, double-blind controlled trial)	20 mL of date vinegar/day	10 weeks	Diabetic participants (n = 60)	- Improved total blood cholesterol, glycated hemoglobin, fasting blood glucose, alanine transaminase, alkaline phosphatase, and folate.	[127]
Antidiabetic (type 2 diabetes) (randomized, placebo controlled clinical trial)	20 mL of red date vinegar/day	10 weeks	Diabetic participants (n = 50)	- Significant reduction in HbA1c, LDL cholesterol, and fasting blood sugar.	[21]
Prevent iron deficiency (anemia) (quasi experimental)	7 dates and iron Supplementation	30 days	Anemic participants (n = 68)	- Showed higher haemoglobin levels.	[141]
Prevent iron deficiency (anemia) (pilot randomized	7 dates/day	4 weeks	Anemic adolescent participants	- Date can be used to manage anemia.<br>- Positive effect on bowel movement.	[142]

controlled trial)			(n = 40)		
Prevent iron deficiency (anemia) (preexperimental)	Not reported	25 days	Anemic adolescent participants (n = 35)	- Increased hemoglobin levels.	[143]
Antihyperlipidemic (randomized, placebo controlled trial)	30 mL of date vinegar/ day	7 weeks	hypercholesterolemic participants (n = 56)	- Improved total cholesterol, triglycerides, LDL and HDL cholesterol, apolipoprotin B.<br>- Enhanced inflammatory biomarkers (C-reactive protein, nitric oxide, tumor necrosis factor alpha, and fibrinogen).	[126]
Antihyperlipidemic (double blinded randomized placebo-controlled study)	30 mL of date vinegar/ day	8 weeks	Hypercholesterolemic participants (n = 76)	- Reduced intestinal lipid absorption.<br>- Improved several serum lipid profile parameters and inflammatory biomarkers.<br>- Increased levels of polyphenols, highly polar carotenoids and fibers.	[125]
Gastrointestinal effect (randomized, controlled, single-blinded, cross-over intervention trial)	7 dates (50g) for the intervention group and 37.1 g maltodextrin–dextrose for the control group	21days followed by 14 days washout period	Healthy participants (n = 22)	- Increased the bowel movements and stool frequency.<br>- Reduced the stool ammonia concentration.<br>- Reduced genotoxicity in human faecal water relative to control.<br>- May reduce colon cancer risk without inducing changes in the microbiota.	[144]
Anticancer (non-randomized controlled clinical trial)	3 dates (7.33 g) and standard treatment	Not reported	Pediatric cancer patients (n = 56)	- Enhanced the treatment outcomes.<br>- Reduced hospital admissions for fever-related neutropenia and infections.<br>- Higher survival rates compared to the control group.	[118]
Anti-inflammatory (quasi-experimental)	2.5 g of date seed in 250ml of water/ day	14 days	Healthy participants (n = 30)	- Reduced pro-inflammatory cytokines and cyclooxygenase (COX) expression.<br>- Decreased the level of interleukin-1, transforming growth factor-, COX-1 and 2.	[119]
Anti-inflammatory (double blind, randomized,	26 g/d date seed powder or placebo for	14 days	Healthy participants (n = 36)	- Decreased oxidative stress, inflammation, muscle pain.<br>- Improved mental health and performance.	[145]

placebo-controlled trial)

ALT: Alanine aminotransferase; AST: Aspartate aminotransferase; ALP: Alkaline phosphatase; NF-κB: Nuclear Factor Kappa B; TNF-α: Tumor Necrosis Factor alpha; IFN: Interferon; JAK1: Janus Kinase 1; STAT3: Signal Transducer and Activator of Transcription 3; MDA: Malondialdehyde; MPO: Myeloperoxidase; CAT: Catalase; SOD: superoxide dismutase; GSH: glutathione; GST: glutathione S-transferase; MTT: 3-(4,5-dimethylthiazol-2-yl)- 2,5 diphenyltetrazolium bromide; HSF: Normal somatic cells; HepG-2: Hepatoma; Caco-2: Colon carcinoma; MDA: Breast carcinoma; AD: Alzheimer's disease; MMP-2: Matrixmetalloproteinase-2; IL: Interleukin.

Table S2. Summary of different studies about the industrial food applications of date palm and its by-products.

Food product application	Nature of date	Concentration	Product type	Main Outcome	Reference
**Bakery product**	Date fruit fiber / date seed hydrolysate and date seed flour	2–5%	Muffins	- Increase in total dietary fiber and ash content.<br>- Increase in biological activities.<br>- Improves texture.<br>- Overall acceptance.	[198,199]
	Date press cake	10%	Biscuit	- Improve sensory and nutritional quality (higher moisture, protein, fiber, minerals, but lower ash and fat content).<br>- Enhance storage stability and recommended dietary allowance.	[170]
	Date fruit flour	15, 17.5, 20,22.5, 25, and 30%	Biscuit	- Higher in crispiness.<br>- Lower the spread ratio.<br>- Higher acceptability at 30%.<br>- Richness in protein. carbohydrate, fat, ash and crude fiber.<br>- Higher shelf life.	[200]
	Date fruit flour	5, 7, 9, and 11	Biscuit	- Improve texture properties (higher hardness).<br>- Increase in polyphenols, fiber contents and antioxidant activity.<br>- Best acceptability at 7 and 9%.	[201]
	Date fiber	5, 10, and	Biscuit	- Significant hypoglycemic effects and anti-obesity actions.	[202]

	15%		- Improve in lipid profile, kidney and liver functions of the obese rats (lower levels of glucose, and cholesterol).	
Date powder	25, 50, and 100%	Biscuit	- Increase in the content of fiber, polyphenols, and antioxidant activity.<br>- Slow down the process of rancidity.<br>- Extend the shelf life.<br>- Acceptable sensory properties.	[203]
Aqueous extract and fiber-rich extraction residue of defatted date seed powder	2.5, 5, and 7.5%	Biscuit dough	- Significant impact on the rheological properties.<br>- Increase in viscoelastic properties of the dough.<br>- Highest resistance to deformation at 7.5%.<br>- Increase in phenolic compounds and antioxidant activity.<br>- Darkness color and increased hardness.<br>- Retarded lipid oxidation during storage and enhanced phenolic retention.<br>- Increased bioaccessibility of polyphenols upon simulated *in vitro* digestion.	[204–206]
Date fruit fiber	5, 10, and 15%	Biscuit	- Improved the sensory characteristics with the best overall acceptability at 10%.<br>- Enhance physical characteristics mainly with 5% and 10%.<br>- Increase in phenolics and antioxidant activity.	[207]
Date seed flour (as a fat substitution)	10, 20, 30, and 40%	Biscuit	- Increase in phenolics and antioxidant activity.<br>- Best sensory acceptability and desired texture was shown at 20%.<br>- High contents of ash, crude fibers, and proteins.	[208]
Date seed	2.5–7.5%	Cookies	- Increase in antioxidant activity, total phenolic content and flavonoids.	[209]
Date seed	5, 10, and 15%	Cookies	- Enhance in nutritional value by significant increase in fiber content.<br>- Decrease in moisture content which extends shelf life.<br>- Sensorial acceptability.	[189]
Date fruit pulp	10, 20, 30, 40, and 50%	Cookies	- Improved the properties of the flours (swelling index, oil absorption capacity, pH and viscosity).<br>- Increased proximate composition except for carbohydrate and protein.	[210]

			- Increased physical properties except for break strength.	
Date fruit powder (as a sugar substitute)	10, 20, 30, 40, and 50%	Cookies	- Enhance the sensory properties.<br>- Improve the physicochemical, antioxidant and mineral content (high in protein, fiber, and ash content).	[211]
Date syrup	10–60%	Cookie	- Decreased hardness.<br>- Lower fracturability.<br>- Darker cookies.	[212]
Date press cake	5, and 10%	Cookies	- Highest score for appearance, texture, flavor, and overall acceptability scores with 10%.<br>- Higher content of health-promoting dietary fiber content.<br>- Darkness color.	[213]
Date seed flour	10, and 30%	Cookies	- Increase in phenolic compounds and antioxidant activity.<br>- Increase in fiber content.<br>- Darkness color.<br>- Similar sensorial and textural properties to control.<br>- Good consumer acceptability.	[214]
Date syrup	50, 75, and 100% (as sucrose substitute)	Sponge cake	- Best level on a sensory-hedonic scale at 100%.<br>- Higher value of total phenolic compounds and antioxidant activity.<br>- Stability in texture properties.<br>- Darkness color.	[171]
Date seed	1–3%	Bread	- Enhancement of dietary fiber content.	[164]
Date seed powder	5, 10, 15 and 20%	Pit bread	- High amount of phenolics and antioxidant activities.<br>- Reduce acrylamide level and increase in fiber content.	[169]
Defatted date seed powder	2.5–5%	Bread	- Increase in dietary fiber.	[215]
Date pulp fiber	2%	Bread	- Increase in dietary fiber.	[216]

			- Improves the physicochemical properties (higher water absorption, stability, tenacity, and smaller extensibility, softening, breakdown and setback).	
Date fruit pulp	100:0, 75:25, 50:50, 25:75, and 0:100 (sucrose: date)	Bread	- Increase in the level of protein, dietary fiber and ash contents.<br>- Lower in carbohydrate level.	[168]
Date seed (Sourdough fermented date seed)	20%	Bread	- Improve sensory aspects.<br>- Reduce bread staling.	[217]
Date fruit pomace (high-fibre dietary)	5, 10, 15, and 20 %	Bread	- Increase fibre and moisture contents accompanied by lower levels of carbohydrates.<br>- Acceptable sensory evaluation.	[218]
Date pomace (As flour Complement)	2.5, 5, 7, and 10%	Pasta	- Increased energy and fiber content.<br>- Reduced cooking time, adhesiveness, and extensibility.<br>- Enhanced swelling index, cooking water absorption, water activity, firmness, and tenacity of pasta.<br>- Most acceptable organoleptically at 2.5%.	[219]
Date syrup	25, 50, 75, and 100% (instead of sugar)	Flakes	- Improved in nutritional quality and potential health benefits.<br>- Overall consumer acceptance.	[220]
Date press cake	10%	Protein bar	- Showed higher flavor, taste, texture, and overall acceptability scores, but lower in color.<br>- Revealed higher protein, fiber, ash, and minerals contents with lower fat and carbohydrates content.	[170]

				- Enhanced storage stability.<br>- Higher willingness to buy.<br>- Higher hardness and cohesiveness.<br>- Gumminess and chewiness are low.	
	Date pomaces	10%	Cereal bars	- Overall acceptability.<br>- Increase in fiber content and high energy<br>- Low water activity.<br>- Darker color.<br>- Texture parameters comparable to the control.	[221]
	Date paste	40, 50, 60, and 70%	Snack bars	- Overall acceptability at 50%.<br>- Improve the textural, sensory and technological qualities.<br>- High in fiber content.	[222]
	Date paste	64%	Energy- rich protein bars	- Showed antihypertensive activity.<br>- Richness in crude fiber and protein.	[223]
	Date paste	49.5 and 34.5%	Bars	- Sensory acceptable.<br>- Stability in physicochemical parameters during storage.	[224]
**Beverage**	Date seed	/	Coffee-like brew	- Considered as bioactive drink and used as therapeutic beverage.<br>- The physicochemical, quality, and sensory attributes were found to be affected by roasting time and temperature.	[186]
	Date seed	/	Coffee-like beverages	- Sensorial Slightly lower sensorial quality compared to Arabica coffee: lighter color, less cloudiness and bitterness, weaker coffee flavor, lower total phenolic content, and reduced antioxidant activity.<br>- Recommended for individuals seeking the characteristic coffee flavor without increasing daily caffeine intake.	[184]
	Roasted date seed powder	/	Coffee	- Source of nutrients (fatty acids, vitamins, and minerals) and polyphenols with high antioxidant potential.	[185]

				- Low cytotoxicity.<br>- Excellent decaffeinated beverage.	
	Date seed	50 and 100%	Coffee	- Showed a volatile profile similar to Arabica coffee.<br>- Rich in bioactive compounds with higher antioxidant activities.<br>- The freeze-drying process was proven to preserve the volatile compounds and increase their solubility.	[187]
**Fermented product**	Fermented date fruit puree	30%	Functional dietary supplement	- Concentration of γ-amino butyric acid, conjugated fatty acids, and insoluble dietary fibers.	[47]
	Date fruit	/	Fiber concentrate	- High antiradical activity.<br>- Have a pleasant chocolate/coffee flavor.<br>- Rich in dietary fiber and antioxidants.	[44,198,225]
**Confectionery product**	Date press cake	9%	Jam	- Enhanced phenolics content and antioxidant activity.<br>- Enhanced the texture profile and microstructure characteristics.	[182]
	Date fruit paste	25, 30, 35, 50, and 40%	Jam	- High content of bioactive compounds (phenolics, flavonoids, and β-carotene).<br>- Overall acceptability.	[226]
	Date seed fiber	1–5%	Chocolate spread	- Overall sensory acceptability.<br>- High-quality functional chocolate spread achieved with dietary fiber enrichment up to 5% without adverse effects.	[227]
	Date fruit paste	25, 50, 75 and 100%	chocolate spread	- Enhanced the crude protein, ash, crude fiber and mineral content.<br>- Overall acceptability (highest preference at 50% for taste, appearance, mouthfeel, and spreadability).	[228]
	Date seed	5, 10, and 15%	chocolate sauce	- Sensorial acceptability.<br>- Decrease in a viscosity.<br>- Extended shelf life due to moisture content reduction.	[189]
	Date syrup	25%	Dark chocolate	- Better physicochemical characteristics than control.<br>- Well accepted sensory evaluation.	[229]

	Date paste (DP)/ date syrup (DS)	50% (DP) and 6.5% (DS)	Protein bars	- Increase in ash, vitamins, fiber, mineral, and amino acids contents.<br>- Better sensory evaluation.	[188]
	Date fruit	1–10%	Candy	- Improved in the nutritional properties.<br>- Positive effect on the functional, phytochemical, and antioxidant properties.<br>- Decreased starch content.	[230]
**Dairy product**	Date press cake	2–6%	Yogurt	- Improved in texture profile.<br>- Enhanced in physical, functional, and sensory properties.<br>- The highest level of overall sensory acceptability was achieved at 2%.	[231]
	Date liquid sugar	1–9%	Yoghurt milk	- Increase in phenolic compounds and antioxidant activity.<br>- Highest scores and firmness, and lowest syneresis at 6%.	[232]
	Date juice	2, 5, and 10%	Bio-yogurt	- Improved the physicochemical properties.<br>- Enhanced the survivability of probiotic bacteria.<br>- Improved diet intake, body weight, haematological, and serum biochemical parameters with 10%.	[174]
	Date paste (DP) and date fruit flour (DF)	3 and 6%	Goat milk yogurt	- Exhibit higher levels of minerals with increased glucose and lactic acid content.<br>- Boost the growth and stability of the yogurt starter culture by enhancing the probiotic potential.<br>- DP reduced the syneresis of yogurts maintaining their physicochemical quality.<br>- DF induces more pronounced modifications than DP.<br>- Overall acceptance.	[177]
	Roasted date seed powder	1, 2, and 3%	Goat yogurt	- Lower acidity and moisture content.<br>- Higher apparent viscosity.<br>- Reduced syneresis.<br>- Increase in protein and mineral contents.<br>- Reduction of aroma and flavor goat milk.	[233]
	Date syrup (DS)/ date fruit	16% with the	Dairy dessert	- Function as a natural thickening agent.	

powder (DP)	rates: Powder DP/DS= 2; DP=DS, and DP=0.5 DS		- Improved apparent viscosity and instant exudation.<br>- Improved antioxidant activities.<br>- Increase in sweetness and antioxidant values.	[234]
Date syrup (DS) and date powder (DP)	14% (DS) and 2% (DP)	Dairy dessert	- Enhance antioxidant capacity, polyphenol content, and composition (lipids, proteins, and dry matter).<br>- Improve microbiological quality.	[172]
Date syrup	4, 7, and 10%	Prebiotic chocolate milk	- Sensory acceptability at higher concentrations.	[235]
Date extract	4, 8, and 12%	Probiotic fermented milk	- Improve physicochemical and rheological characteristics.<br>- Higher acidity and antioxidant potential.<br>- Lower pH and syneresis.<br>- Similar sensory acceptability.	[236]
Date paste	10, 20, and 30%	Fermented milk	- Improved the nutritional composition, viscosity, and microbiological quality.<br>- Milk with 10% and 20% had higher acceptable sensory.	[237]
Date fruit powder	10, 15, 20, and 25%	Milk	- Considerable increase in dietary fibers and antioxidant content.<br>- Nutritional quality stability at 25%.	[176]
Date Syrup	2.5, 5, 7.5, 10, 12.5, and 15%	Fermented milk	- Fortified drink with 12.5% improve nutritional quality (increase ash, protein, sugars, and magnesium).<br>- Increase the taste and overall acceptability).<br>- Decrease in total microbial count, and total yeast and molds count.<br>- Maintain the nutritional, microbial, and sensory quality properties during storage.	[238]
Date fruit pasta	0, 5, 7.5, 10, 12.5, and 15%	Fermented camel milk	- Improved the nutritional quality (ash, dietary fiber, minerals, carbohydrate content, phenolic compounds and antioxidant activity).<br>- Enhanced flavor, texture, consistency, and mouthfeel.	[239]

				- Increased probiotics viability (bifidobacterial).	
	Date syrup	6 and 8%	Bio-Fermented Camel Milk	- Increase in ash, minerals, acetaldehyde, total phenolic contents, and titratable acidity, viscosity, and antioxidant.<br>- Increased the total bacterial and bifidobacteria counts.<br>- Improved the sensory scores for flavor, consistency, appearance.	[175]
	Date seed	5, 10, 15, and 20%	Cheese	- High sensory acceptability at 5% and 10%.<br>- Increase in nutritional value.<br>- Affect Negatively the texture.	[173]
	Date seed	1, 5, and 10%	Spreadable processed cheese	- Increase in nutritional value (total solids and fiber contents).<br>- Significant effect on texture, sensory properties.	[240]
	Date paste	4 and 8%	Cheese	- Exhibited a higher relative proportion of flavonols than the original date paste.<br>- Reduced the stability of soluble-free polyphenols.	[241]
**Meat product**	Date paste	2.5 and 7.5%	Spreadable liver pâtés	- Improve chemical composition and enhance physicochemical characteristics (pH, aw, color, emulsion stability, and texture).<br>- Showed an acceptable sensory quality.<br>- Reduce oxidation and microbial growth.	[242,243]
	Date paste	5,10, and 15%	Pork liver pâté	- Increase color stability during storage.<br>- Protected against lipid oxidation during storage.<br>- Overall acceptance.	[244]
	Date seed extract	4%	Camel meat sausages	- High in hardness, gumminess, and chewiness on day 12 of storage.<br>- No influence on sensory color over 12 days of storage at 4°C.<br>- Overall acceptability.	[245]
	Date paste	5%	Paprika dry-cured sausage	- Overall acceptability.<br>- Physicochemical properties similar to the traditional one.<br>- Positively sensory properties evaluated.	[246]

	Date seed powder	20%	Beef burger	- Enhance in moisture, protein, ash, Ca, and Na content as well as the cooking properties and greater softness.<br>- Exhibit greatest lightness, redness, and yellowness scores.<br>- Effectively used as a fat substitution in meat manufacturing.	[247]
	Date seed	1.5, 3, and 6%	Beef burger	- Improve the shelf life and the cooking properties.<br>- Enhance stability of the color, lipid oxidation, and microbial growth during the storage time.<br>- Improve the composition of bioactive compounds.	[180]
	Date powder (at first stage of maturity)	25, 50,75, and 100%	Beef burger	- Preserve the red color of raw burgers and improve the cooking properties.<br>- Avoid pigment and lipid oxidation.<br>- Reduce microbial counts.<br>- Shown sensory acceptability.	[248]
	Date pomace fibers	2.5, 5, 7.5, and 10%	Ground patty analogues	- Increased fibrousness and contributed to a more stable internal structure.<br>- Contributed to color by imparting brown–red tones, resulting in an appearance closer to conventional beef patties.	[179]
	Date seed extract	0.156, 0.312, and 0.624%	Chicken breast meat	- Inhibits microbial growth.<br>- Reduces chemical. alterations (lowered lipid and protein oxidation).<br>- Improves sensory attributes.<br>- Increased shelf life by over 14 days at 0.6%.	[249]
**Food packaging**	Date syrup waste extract	5, 10, 15, and 25%	Active gelatin film	- Enhanced the gelatin-water interactions (improve water solubility and moisture content).<br>- Lower tensile strength and high flexibility at 25%.<br>- The active film with 25% showed a relatively higher water vapor permeability and inhibitory effect on oil thermos-oxidation.<br>- Exhibited a high release profile of the active phenolic compounds and superior antioxidant capacity.	[194]

			- Decrease in light transmission with film at 25%.	
Date seed powder	5, 10, 15, and 20%	Carboxymethyl cellulose (CMC) based edible film	- Increased thickness.<br>- Reduced the solubility.<br>- Exhibited antibacterial activity at 15 and 20%.<br>- Increase in the antioxidant capacity with increasing concentration.<br>- Improved in the water vapor permeability.<br>- Decreased tensile strength.<br>- Increased elongation at break.	[192]
Date seed powder	10, 20, 30, and 40%	Biodegradable film	- Increased thickness, tensile strength, Young's modulus, phenolic compounds, and antioxidant activity.<br>- Decreased in swelling index, water solubility, water vapor permeability and elongation capacity.<br>- Improved the morphological properties of the films up to 30%.	[191]
Date seed extract	2., 2.5, 3.5, 4.5, and 7%	Active nanocomposite films	- Decreased mechanical properties of the film.<br>- Increased antibacterial activity with increasing the extract concentration.<br>- Optimal formulation for highest antibacterial properties was found to be 4.1 %.	[250]
Date syrup waste extract	15%	Active composite Films	- Exhibited high active compounds migration to aqueous food simulants.<br>- Increase water content, solubility, water vapor permeability, tensile strength and Young's modulus.<br>- Higher phenolic compounds rates and antioxidant activity.	[197]
Date seed oil	0.29, 1, 1.71, and 2%	Composite film	- Conferred good light barrier properties to the films.<br>- Positive effect on water barrier properties.<br>- Improved light barrier properties.<br>- Higher thermal stability.	[251]
Date seed powder	5, 10, 15, and 25%	Biodegradable hybrid polymer	- Faster biodegradation rate.<br>- Higher water absorption.	[252]

		composite	- Displayed higher tensile strength.	
Date seed oil	0.5, 1, 1.5, and 2%	Edible wax coating	- Extended the shelf stability of guava fruit.<br>- Improve the attributes of pH, weight loss, Total solid soluble, titratable acidity, fruit firmness, and vitamin C.	[193]
Date seed extract	0.22, 0.44, and 0.88 %	Active composite film	- Improved the physical, mechanical, thermal and optical properties of the film.<br>- Improved the antioxidant and antibacterial activities.<br>- Enhanced goat meat quality and safety by minimizing color changes, preventing metmyoglobin formation, and delaying lipid and protein oxidation as well as microbial growth during storage.	[253]
Date seed	10, 20, 30, and 40%	Active Alginate-based film	- Improved water vapor barrier properties.<br>- Increased tensile strength and elongation at break.<br>- Lowest loss of phenolic content and antioxidant activities after storage for 3 months with 40%.<br>- Exhibited good light barrier properties.<br>- Exhibited highest physical and mechanical properties and good antioxidant and light barrier properties with a range of 10%–20%.	[254]
Date fruit powder extract	5, 10, and 15	Edible composite film	- Enhance thermal stability.<br>- Modest amount of flexibility with good tensile strength.<br>- Films' antimicrobial qualities were superior against tested foodborne pathogens.<br>- Maintained the quality and freshness of strawberries packed during 10 days at 25°C.	[195]
Date seed extract	2, 4, and 8%	Active composite film	- Revealed superior antimicrobial possibilities at 8%.	[255]
